
JOURNAL INFORMATION
==============================
NLM Title Abbreviation: ScientificWorldJournal
Journal ID: ScientificWorldJournal
NLM Journal ID: 101131163
Journal ID: TSWJ

The Scientific World Journal

ISSN: 2356-6140
EISSN: 1537-744X
Publisher: Wiley

ARTICLE INFORMATION
==============================
PMCID: PMC5956402
PMID: 15349564
DOI: 10.1100/tsw.2004.91
Article ID: 383049
Article version: 1
Subjects: Research Article

Surgery Of The Adrenals

Vaughan E. D.
Colt James J.
The James Buchanan Brady Foundation, The New York Hospital-Cornell University Medical Center, New York, NY, USA
Publication date: 2004
Electronic publication date: 2004 Jun 28
Volume: 4
First page: 400
Last page: 426
Copyright: Copyright © 2004 E. D. Vaughan and James J. Colt.
Copyright year: 2004
License: This is an open access article distributed under the Creative Commons Attribution License, which permits unrestricted use, distribution, and reproduction in any medium, provided the original work is properly cited.
License URL: https://creativecommons.org/licenses/by/3.0/

==============================
Article
*TheScientificWorldJOURNAL* (2004) 4 (S1), 400–426
ISSN 1537-744X; DOI 10.1100/tsw.2004.91

### Surgery Of The Adrenals

E. Darracott Vaughan, Jr., M.D. and James J. Colt, Professor of Urology
*The James Buchanan Brady Foundation, The New York Hospital-Cornell University Medical Center, New York, New York*

**E-mails**: evaughan@med.cornell.edu

*Previously published in the Digital Urology Journal*

**DOMAIN**: urology

### INTRODUCTION

The major adrenal tumors which will be discussed in this presentation include adrenal cortical adenomas producing primary hyperaldosteronism and Cushing's Syndrome, adrenal cortical carcinoma, incidentally identified adrenal masses and pheochromocytoma. Actually the most common tumors involved in the adrenal glands are metastatic tumors to the adrenal, the management of such lesions generally is dependent upon the treatment of the primary disease entity. It is fortunate that the diagnosis of the most common adrenal disorders is extremely accurate using a combination of precise analytic methods for the measurement of the abnormal secretion of adrenal hormones and sophisticated radiographic techniques for the localization and characterization of specific adrenal lesions.

The current article relies heavily on tables and figures excerpted from other more comprehensive chapters and the interested reader is guided to those texts for a more comprehensive discussion of these disorders.[1-4]

The management of patients with adrenal disorders requires a clear understanding of the normal physiology of the adrenal, medulla and cortex, a three-dimensional concept of the adrenal anatomy as well as adjacent structures, and the knowledge of the various pathologic entities which may involve the adrenal. Currently, the hormonal evaluations to delineate the different disorders have been standardized and are shown on the enclosed flow charts. Moreover, common errors which can occur during the attempted diagnosis of these disorders are outlined in Table 1.

Once the appropriate diagnosis has been made, the surgeon should be knowledgeable of the pre-operative patient preparation (Table 2) and the various surgical techniques (Table 3) as well as the option of laparoscopic adrenalectomy and also be aware of potential complications which can occur during surgery.

Finally, the operative and post-operative complications which can occur are both generic to major urological surgery or specific based on the underlying adrenal disorder (Table 4, Table 5). Accordingly this presentation reviews the pre-, intra- and post-operative aspects of each of these specific entities and will outline surgical approaches with operative hints to guide those interested in adrenal surgery.

**TABLE 1**
**Pitfalls in the Diagnosis of Surgical Adrenal Disorders**

**Primary Aldosteronism**

Challenge with sodium loading (10 gm/day) before measuring plasma $K^+$.

Repletion of $K^+$ to normalize plasma $K^+$ before measuring plasma or urinary aldosterone.

Complete reliance on a postural aldosterone stimulation test (70% accuracy).

Failure to measure cortisol during adrenal vein sampling of aldosterone to validate correct positioning.

Failure to recognize bilateral adrenal hyperplasia.

Adrenal hemorrage during adrenal vein sampling.

**Cushing's Syndrome due to Adrenal Adenoma or Carcinoma**

Failure to identify the use of exogenous steroids causing Cushing's Syndrome.

Inadequate physical examination essential for the diagnosis..

Knowledge that alcoholism and depression can mildly elevate plasma cortisol (pseudo-Cushing's).

Inability to diagnose pituitary Cushing's by finding elevated plasma ACTH (corticotrophin).

**Adrenal Carcinoma**

Evaluation for metastatic disease.

**Incidentaloma**

Metabolic evaluation to identify functional lesions.

MRI to determine tissue composition

**Pheochromocytoma**

Careful evaluation to reveal multiple lesions.

Measurement of urinary catecholes and metabolites even if plasma catecholes are normal.

Evaluation for other components when Multiple Endocrine Abnormailty (MEA) syndromes are suspected.

The adrenal glands are paired retroperitoneal organs that lie within the perinephric fat, at the anterior, superior and medial aspects of the kidneys. Their location in juxtaposition with other organs as well as the periadrenal fat renders them ideal for sectional imaging by computer tomography (CT). Thin-cut CT scanning allows precise identification of lesions as small as 0.5 cm. The CT scan remains the best imaging device for the identification of small adrenal lesions whereas magnetic resonance imaging (MRI) gives information concerning cell type and aids in the differentiation of adenomas from medullary tumors or metastatic carcinoma.[5] Other advantages of MRI scanning will be discussed later. The right adrenal lies above the kidney posterior and lateral to the inferior vena cava (IVC) and its solitary venous drainage is via a short stubby vein that enters the IVC in a posterior fashion. Hence the right adrenal gland is the best approached through a posterior or modified posterior incision.[6] The left adrenal is in more intimate contact with the kidney overlying the upper pole of the kidney with its anterior surface and medial surface behind the pancreas and splenic artery. It is best exposed through a flank approach or a thoraco-abdominal approach if the lesion is large.

**TABLE 2**
**Errors in Patient Preparation for Adrenal Surgery**

**Primary Aldosteronism**

- Potassium repletion
- Blood pressure control

**Cushing's Syndrome**

- Inhabition of glucocorticoid production when there are severe manifestations using metapyrone.
- Control of diabetes
- Pre-operative steroid administration
- Operative steroid administration

**Incidentalomas**

- Anesthetic preparation for pheochromocytoma - 5% have negative diagnostic studies.

**Adrenal Carcinoma**

- Consent for adjacent organ removal
- Failure to identify IVC involvement

**Pheochromocytoma**

- Pre-operative catechole blockade
- Volume expansion
- Anesthesia consultation

The adrenals have a delicate, rich blood supply estimated to be 6-7 ml/gm/min without a dominant renal artery. The inferior phrenic artery is the main blood supply with additional branches from the aorta and renal arteries. The small arteries penetrate the gland in a circumferential stellate fashion leaving both the anterior and posterior surfaces avascular (Fig. 1). During adrenalectomy an important technical goal is to divide the superior and lateral blood supply to the adrenal first, allowing the adrenal to remain attached to the kidney which can be used to draw the adrenal gland inferiorly and anteriorly during the resection. On the left side, the adrenal vein drains into the left renal vein, however there is also a medially located phrenic drainage branch which if not appropriately ligated can cause troublesome bleeding (Fig. 2). The left adrenal vein is also a guide to the left renal artery which often lies dorsal to the vein and one potential complication of left adrenalectomy is the inadvertent ligation of the apical renal arterial branch to the upper pole which lies in close contact to the inferior border of an adrenal tumor.

The basic physiology of the adrenal cortex and medulla as well as the various pathological entities will be discussed under specific disorders.

### CUSHING'S SYNDROME

Cushing's Syndrome is the term utilized to describe the symptom complex caused by excessive circulating glucocorticoids. We must remember that the term is all encompassing and includes patients with pituitary hypersecretion of ACTH (corticotropin), Cushing's disease, which accounts for 75-80% of patients with endogenous Cushing's, patients with adrenal adenomas or carcinomas, and patients with ectopic secretion of ACTH or corticotropin releasing hormone (CRH syndrome).[7] Before assuming a

**TABLE 3**
**Surgical Options**

| Disease | Approach |
|---|---|
| Primary hyperaldosteronism | Posterior (left or right)<br>Modified posterior (right)<br>11th Rid (left>right)<br>Posterior Transthoracic |
| Cushing's adenoma | 11th Rib (left or right)<br>Thoracoabdominal (large)<br>Posterior (small) |
| Cushing's disease | Bilateral posterior<br>Bilateral 11th Rib (alternating) |
| Adrenal carcinoma | Thoracoabdominal<br>11th Rib<br>Transabdominal |
| Bilateral adrenal ablation | Bilateral posterior |
| Pheochromocytoma | Transabdominal Chevron<br>Thoracoabdominal (large-usually right)<br>11th Rib |
| Neuroblastoma | Transabdominal<br>11th Rib |

(From: Vaughan ED Jr: Adrenal surgery, in Operative Urology, F Marshall (ed). W.B.Saunders, 1991.)

patient has one of these pathological entities there should be a thorough questioning of the patient about the use of steroid containing preparations. At times patients are unaware that a substance they use, particularly creams or lotions, contain steroids and if the patient is on any type of medication at all, it should be carefully reviewed for steroid content. There are few diseases in which the clinical appearance of the patient can be as useful in suspecting the diagnosis. Old photographs are helpful in documenting recent changes in appearance that occurred. The more common clinical manifestations of Cushing's Syndrome found in different series of patients is shown in Table 6. The clinical findings do not distinguish patients with Cushing's disease from those with adrenal adenoma however, patients with adrenal carcinoma are more likely to show virilization in the female or feminization in the male. Patients with ectopic ACTH may present with manifestations of the primary tumor. It is also important to remember that some non-endocrine disorders mimic the clinical and even the biochemical manifestations of Cushing's Syndrome. These patients have been termed to have pseudo Cushing's Syndrome, this may exist in patients with major depression or patients which chronic alcoholism.[7]

There are a myriad of tests to both diagnose the presence of Cushing's Syndrome and then to identify which sub-entity is present. Fortunately, recent extremely accurate assays for urinary and plasma cortisol as well as plasma corticotropin, this task has become much easier. The approach which has recently been reported by Orth is shown in (Fig. 3).[7]

The clinical diagnosis of Cushing's Syndrome is confirmed by the demonstration of cortisol hypersecretion. At the present time the determination of 24 hour urinary excretion of cortisol in the urine is the most direct and reliable index of cortical secretion. Orth recommends that urinary cortisol should be measured in 2 and preferably 3 consecutive 24 hour urine specimens, collected on an out-patient basis.

**TABLE 4**
**Adrenal Surgery Operative Complications**

**Hemorrhage**
- IVC
- Adrenal vein
- Lumbar vein
- Hepatic vein

**Vascular**
- Ligation of renal artery branch
- Ligation of mesenteric artery
- IVC involvement

**Adjacent Organ Injury**
- Pneumothorax
- Pancreas
- Liver
- Spleen
- Stomach
- Colon
- Kidney

Once the diagnosis has been established then the next chore is to determine whether there is Cushing's disease due to hypersecretion of plasma corticotropin (ACTH) from the pituitary or primary adrenal disease. Herein lies the major change in our approach to patients with Cushing's disease. In the past high and low dose dexamethasone suppression tests have been used to accomplish this task. At the present the low dose dexamethasone is generally used to rule out pseudo Cushing's Syndrome. The differentiation of corticotropin dependent Cushing's vs. corticotropin independent Cushing's Syndrome is determined by the concurrent late afternoon or midnight measurement of collection of blood for the simultaneous measurement of plasma corticotropin and cortisol. Thus if the patient's cortisol concentration is > 50 mcg/dl and the corticotropin concentration is < 5 pg/ml, then the cortisol secretion is ACTH independent and the patient has a primary adrenal problem. In contrast, if the plasma corticotropin concentration is > 50 pg/ml then the cortisol secretion is ACTH dependent and the patient has Cushing's Syndrome or ectopic ACTH or CRH syndrome.[7] In situations where the two site immunoradiometric assay test is not available, the high dose dexamethasone suppression test has always been used as the standard test to differentiate between pituitary and adrenal Cushing's Syndrome. Patients are given high dose dexamethasone (2 mg every 6 hrs for two days) and plasma cortisol and urinary free cortisol levels are measured. In patients with pituitary disease, there should be a 50% or greater suppression in cortisol. Patients with adrenal adenomas or carcinomas fail to suppress cortisol secretion. The high dose dexamethasone suppression test may also be useful to identify ectopic ACTH syndrome where there is usually complete resistance to high dose dexamethasone suppression.

**TABLE 5**
**Post-Operative Complications Following Adrenal Surgery**

| | |
|---|---|
| **Primary Aldosteronism** | |
| | Hypokalemia - secondary to continued potassium loss immediately post-operative |
| | Hyperkalemia - secondary to failure of contralateral adrenal to secrete aldosterone |
| **Cushing's Syndrome** | |
| | Inadequate steroid replacement leading to hypocorticoidism. |
| | Fracture secondary to osteoporosis |
| | Hyperglycemia |
| | Poor wound healing |
| | Increased risk of infection |
| **Pheochromocytoma** | |
| | Hypotension secondary to alpha- adrenergic blockade after tumor removal |
| **Generic Complications** | |
| | Hemorrhage |
| | inferior vena cava |
| | adrenal arteries |
| | Pneumothorax |
| | Pancreatitis |
| | Pneumonia |
| | Hiccoughs |

If the patient is identified as having adrenal Cushing's the next step then is radiographic localization with CT scanning.[8] Adrenal adenomas are usually larger than 2 cm, solitary and are associated with atrophy of the opposite gland. The density is low because of the high concentration of lipid (Fig. 4). Adrenal carcinomas are often indistinguishable from adenomas except for the larger size, carcinomas usually being greater than 6 cm.[9] Necrosis and calcification are also more common in association with adrenal carcinoma but are not specific. Clearly large irregular adrenal lesions with invasion represent carcinoma, however metastatic carcinoma to the adrenal has the same appearance.

MRI is not usually necessary in patients with Cushing's Syndrome unless the lesion is large and the rationale for MRI is to obtain anatomical information concerning surrounding structures or invasion of the inferior vena cava, a rare but well recognized entity.[10]

Adrenal cortical scanning with iodinated cholesterol agents is no longer routinely utilized but can be helpful in differentiating functional adrenal tissue from other retroperitoneal lesions.[11]

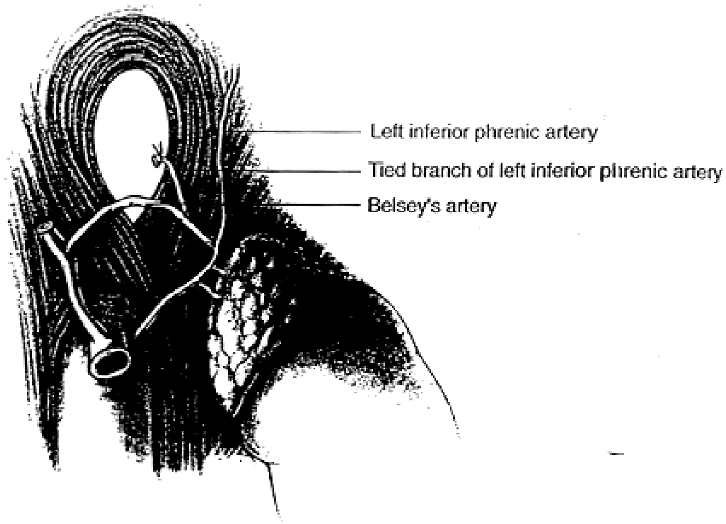

**FIGURE 1**. Superior blood supply to the adrenal showing Belsey's artery which lies quite close to the esophageal hiatus and runs in close contact to the phrenic vein. (From: Skinner DB: Atlas of Esophageal Surgery. Churchill Livingston: NY, 1991, pp. 119).

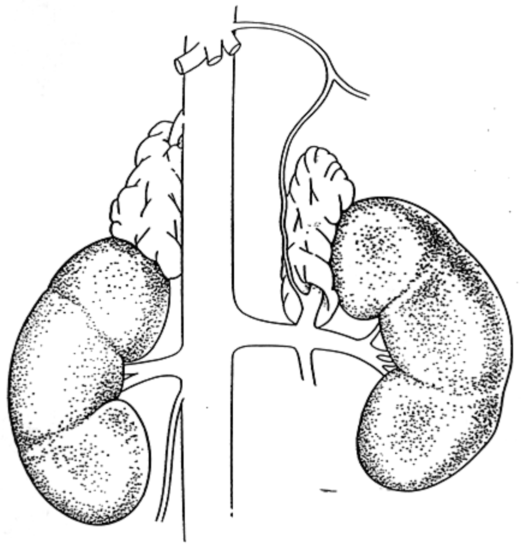

**FIGURE 2.** Venous drainage from both adrenals showing the presence of both the left adrenal vein and the phrenic branch. Also showing the short, stubby posterior arising right adrenal vein.

### INCIDENTALLY DISCOVERED ADRENAL MASSES

The increased utilization of abdominal ultrasound and CT scanning has led to a new classification of adrenal lesions termed the incidentally identified unsuspected adrenal mass or "incidentaloma".[9] Our approach to the incidentally identified adrenal mass is shown in Figure 4. Several points do not warrant controversy. First there is agreement that all patients with solid adrenal masses should undergo biochemical assessment. If biochemical abnormalities are identified the lesions should be treated appropriately as described elsewhere in the chapter, usually by removal of the offending lesion. However, the extent of biochemical evaluation has been reviewed and a selective approach has been outlined which markedly limits cost without sacrificing diagnostic accuracy.[12] A very limited evaluation is recommended including only test to rule out pheochromocytoma, potassium levels in hypertensive cases and glucocorticoid evaluation only the presence of a clinical stigmata of Cushing's Syndrome or virilization. The second point that is non-controversial is that non-functioning solid lesions larger than 5cm should be removed. This is based on the finding that adrenal malignancies are almost always larger than 6 cm. However, we feel that CT scanning may underestimate the size of an adrenal and we suggest that exploration be performed when lesions are more than 5 cm on CT or MRI.[13] Furthermore, if lesions are purely cystic by CT or MRI, cyst puncture is often not necessary and these lesions can be followed. The controversy arises in the management strategy for the solid adrenal lesions, smaller than 5 cm in size. The current approach has been to use MRI imaging in this situation. Most adenomas appear slightly hypo-intense or iso-intense relative to the liver or spleen on T1 weighted images and slightly hyper-intense or iso-intense relative to hepatic or splenic parenchyma on T2 weight. Indeed there is little change in the intensity from T1 to T2 weighted studies. In contrast, the general notion is that adrenal cortical carcinoma is hypo-intense relative to liver or spleen on T1 weighted images and hyper-intense to the liver or spleen

**TABLE 6**
**Clinical Manifestations of Cushing's Syndrome**

| | All[1] % | Disease[2] % | Adenoma/ Carcinoma[3] % |
|---|---|---|---|
| Obesity | 90 | 91 | 93 |
| Hypertension | 80 | 63 | 93 |
| Diabetes | 80 | 32 | 79 |
| Centripetal obesity | 80 | -- | -- |
| Weakness | 80 | 25 | 82 |
| Muscle Atrophy | 70 | 34 | -- |
| Hirsuitism | 70 | 59 | 79 |
| Menstrual abnormal/sexual dysfunction | 70 | 46 | 75 |
| Purple striae | 70 | 46 | 36 |
| Moon Facies | 60 | -- | -- |
| Osteoporosis | 50 | 29 | 54 |
| Early bruising | 50 | 54 | 57 |
| Acne/pigmentation | 50 | 32 | -- |
| Mental changes | 50 | 47 | 57 |
| Edema | 50 | 15 | -- |
| Headache | 40 | 21 | 46 |
| Poor healing | 40 | -- | -- |

[1]Hunt and Tyrell, 1978; [2]Wilson, 1984; [3]Scott, 1973; (From: Scott HW Jr: Surgery of the Adrenal Glands, HW Scott (ed). J.P. Lippincott Co: Philadelphia, 1990).

on T2 weighted images. Thus if the mean signal intensity ratio between the lesion and the spleen is over 0.8, it is unlikely that the lesion is a benign adenoma. However it should be remembered that there are a number of entities other than adrenal carcinoma which can cause high intensity including neural tumors, metastatic tumors to the adrenal, adrenal hemorrhage and other retroperitoneal lesions.[14,15] An additional study which has shown accuracy is the fine needle adrenal biopsy guided by ultrasound or CT. In a large series from Finland, significant cytologic material was obtained in 96.4% and the accuracy to differentiate

**An Approach to the Diagnosis of Cushing's Syndrome and its Cause**

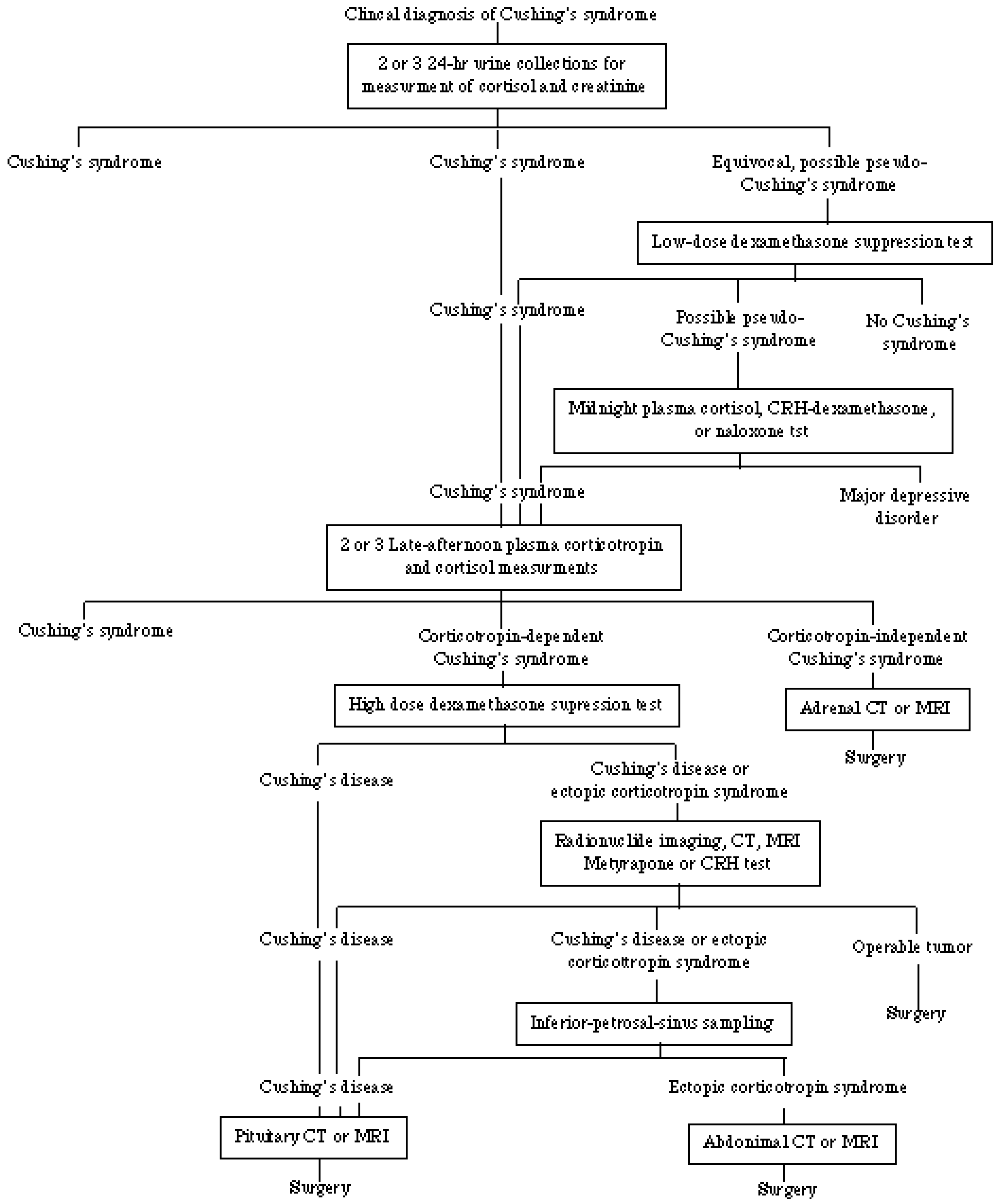

**FIGURE 3**. (From: Orth DN: Cushing's Syndrome. N Eng J. Med., 32:791, 1995.)

benign from malignant disease was 85.7%.[16] However, the utilization of aspiration cytology requires an extremely experienced cytologist and in fact there is often inability to distinguish an adrenal adenoma from a carcinoma even upon pathologic review of the entire specimen.

It is our general approach that if there is either any radiographic evidence which argues against a characteristic benign adenoma or if there is any change in size of an adrenal lesion with repeated studies, then we feel that adrenalectomy is indicated. This fairly aggressive approach is justified in view of the extremely poor prognosis of patients when adrenal carcinoma is diagnosed even when the lesion is localized.

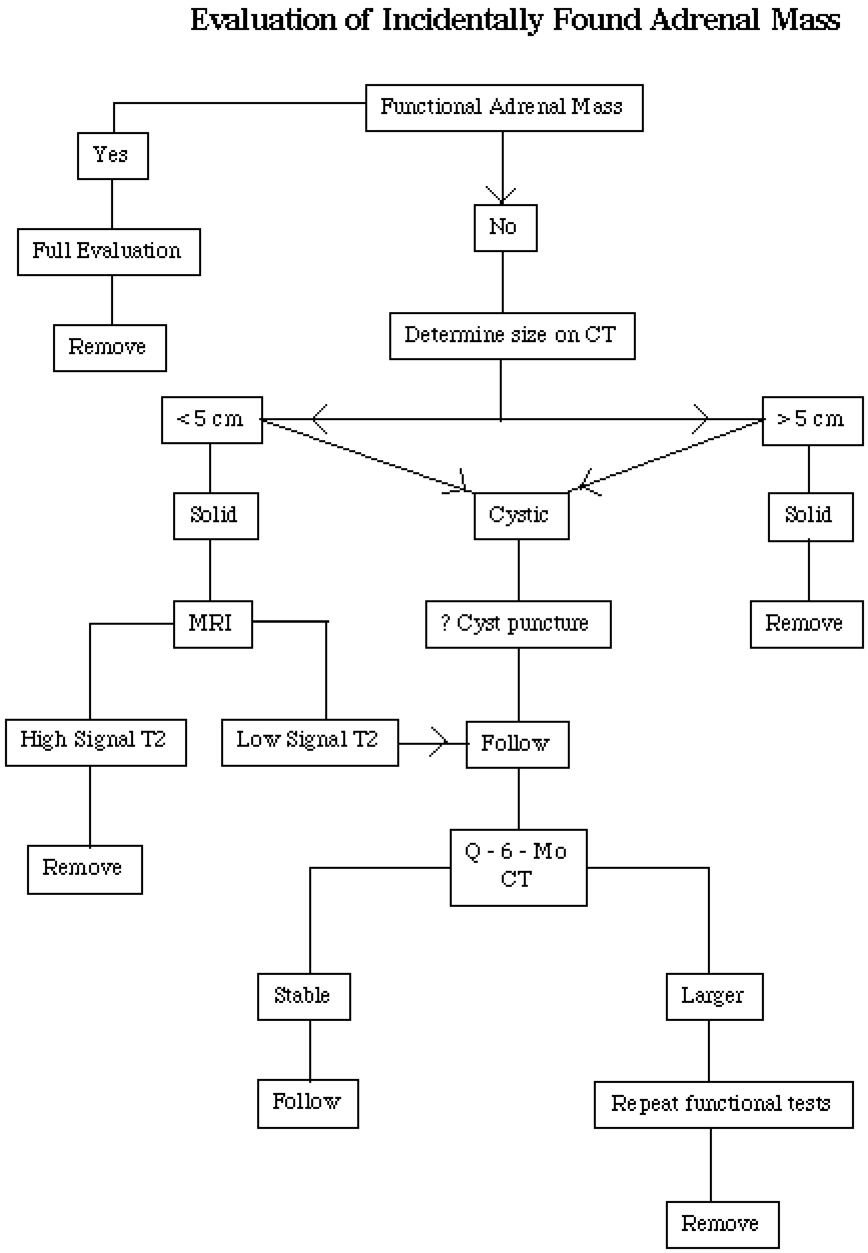

**FIGURE 4**

### ADRENAL CARCINOMA

Adrenal carcinoma is a rare disease with a poor prognosis. The incidence is estimated as one case per 1.7 million accounting for only 0.02% of cancers. A practical sub-classification for adrenal carcinomas is according to their ability to produce adrenal hormones. In a series of Luton and co-workers,[17] 79% of adrenal tumors were functional, a higher percentage than previously reported due to more sensitive assays. The varieties of functioning tumors are shown in Table 7. However, this classification is somewhat contrived since many of these tumors will produce multiple adrenal hormones and also because of the clear evidence that a tumor may secrete one hormone at one point in its natural history and additional hormones at a later phase when there is increased tumor mass. The most commonly identified functional tumor is one causing Cushing's Syndrome. The most common characteristic to delineate Cushing's Syndrome due to carcinoma rather than adenoma has been the presence of virilization with elevated 17-ketosteroid levels. More recently the measurement of DHEA has been useful in identifying these patients.

**TABLE 7**
**Classification of Adrenal Carcinoma**

**Functional**
- Cushing's syndrome
- Virilization in females
  - Increased DHEA, 17-ketosteroids
  - Increased testosterone
- Feminizing syndrome in males
- Hyperaldosteronism
- Mixed combinations of above

**Nonfunctional**

DHEA, dehydroepiandrosterone

Other rare functional tumors include both testosterone and estrogen secreting adrenal cortical tumors. Rarely virilization can occur in the absence of elevated urinary 17-ketosteroids and raises the possibility of pure testosterone secreting ovarian or adrenal lesions.[18] Of the two sites of origin adrenal cortical tumors secreting testosterone are exceedingly rare. In contrast to other tumors described in this section these tumors are usually small, less than 6 cm and many behave in a benign fashion. In contrast, most feminizing tumors occur in males 25 to 50 years of age, they are usually larger, often palpable and highly malignant.[19] Characteristically the patients present with gynecomastia, in addition they may exhibit testicular atrophy, impotence or decreased libido. We have also seen a presentation with infertility and oligospermia. These tumors secrete androstenedione which is converted peripherally to estrogen. Other steroids may also be secreted and the clinical picture may mixed with associated Cushinoid features.

The management of adrenal cortical carcinoma is surgical removal of the primary tumor. The most common sites of metastasis include lung, liver and lymph nodes.[20] Often these tumors extend directly into adjacent structures, especially the kidney and surgical removal may require removal of the primary tumor and adjacent organs including the kidney, spleen as well as local lymph nodes. Unfortunately, despite onbloc resection even in patients without evidence of metastatic disease, the 5 year survival rate is only approximately 50% with complete resection and 35% overall.[21] Because of the poor prognosis there has been an intense search for effective adjunctive chemotherapy, but this search has been frustrating and it is generally believed that conventional chemotherapy is not effective, probably because of p-glycoprotein expression.[22] The most success has been reported with the adrenolytic (1,1-dichloro-2-[0-chlorophenyl]-2-[p-chlorophenyl]-ethane)(o, p -DDD) or mitotane.[23] This DDT derivative has been shown to induce tumor response in 35% in a review of 551 cases reported in the literature.[24] However, despite these response rates, survival time has not been prolonged and there is intense toxicity. Recently it has been suggested that patients even without the presence of metastatic disease be given adjunctive o,p-DDD, and trials are currently in progress to determine if this approach is efficacious.

In general, there is an extremely poor prognosis in patients with adrenal cortical carcinoma and there is obvious need for the development of new treatment strategies.

### HYPERALDOSTERONISM

The term hyperaldosteronism originally was coined by Dr. Jerome Conn to describe the clinical syndrome characterized by hypertension, hypokalemia, hypernatremia, alkalosis and periodic paralysis due to an aldosterone-secreting adenoma.[25] We now realize that this metabolic syndrome can be caused by either a solitary adrenal adenoma or by bilateral adrenal zona glomerulosa hyperplasia. One of the clinical chores is to delineate patients with hyperplasia from those adenoma.[2,26] The syndrome of primary hyperaldosteronism is now identified by the combined findings of hypokalemia, suppressed plasma renin activity (PRA) despite sodium restriction, and a high urinary and plasma aldosterone level after sodium repletion in hypertensive patients. The current evaluation of patients suspected of having hyperaldosteronism is shown in Figure 5. The primary physiologic control of aldosterone secretion is angiotensin II.[27,28] Other control mechanisms are ACTH and potassium. A clear knowledge of the physiology of the renin-angiotensin-aldosterone system is mandatory in order to understand the pathophysiology and evaluate patients with primary hyperaldosteronism. The critical sensor in the renin-angiotensin-aldosterone system (RAAS) resides in the juxtaglomerular apparatus within the kidney. Thus, in response to a variety of stimuli, but primarily decreased renal perfusion, or a decreased intake of sodium, there is an increase renin release, formation of angiotensin II and subsequent aldosterone secretion. Therefore the term secondary hyperaldosteronism is utilized when there is increased renin secretion and secondary aldosterone production. The most common examples of secondary hyperaldosteronism would be renovascular hypertension[29] or malignant hypertension.[30] In contrast, with an adrenal adenoma or adrenal hyperplasia there is primary secretion of aldosterone and subsequently the sodium retention which occurs leads to a suppression of plasma renin activity.

Therefore returning to Figure 5, the hallmark of the entity is hypokalemia. However some patients realize that weakness occurs with increased sodium intake, therefore restrict their sodium, and may have a normal potassium than first observed. Therefore the entity should not be ruled out until the patient has sodium loading with 10 gms of sodium a day for several weeks and repeat potassium measurements. A small subset of patients exhibit normokalemic hyperaldosteronism and if there is a high index of suspicion for the disease, these patients should be studied further. If there is hypokalemia, a 24 hour urine should be collected demonstrating that there is urinary loss of potassium. The critical test is the measurement of plasma of renin activity at a time when the patient is either on a low sodium diet or is challenged with a diuretic. If the patient has hyperaldosteronism the plasma renin activity remains inappropriately low despite sodium depletion. Because potassium is also a stimulus of aldosterone, the patient should be potassium repleted before measuring 24 hour urine and plasma aldosterone levels. Both of these values should be elevated in hyperaldosteronism.

At this point the question is whether the patient has a unilateral adenoma or bilateral adrenal hyperplasia and the imaging study of choice is an adrenal CT scan with 3-5 mm cuts through both adrenal glands. The next step which is traditionally performed would be adrenal vein sampling. The difficulty with adrenal vein sampling is obtaining adequate collections from the short, stubby, right adrenal vein and when samples are collected, cortisol levels should always also be collected to ensure proper catheter placement. An appropriate way of analyzing aldosterone levels is with comparative aldosterone/cortisol ratios from each side. It is our general policy to have positive lateralizing information as well as a positive CT scan before recommending exploration and unilateral adrenalectomy. However, more recently in patients who have elevated plasma 18-hydroxy-B levels and elevated urinary 18-hydroxy-F, 18-oxy-F levels at times we have not required sampling when a clear adenoma was demonstrated on CT scan. In contrast, we have demonstrated a subset of patients with radiographic bilateral hyperplasia who will

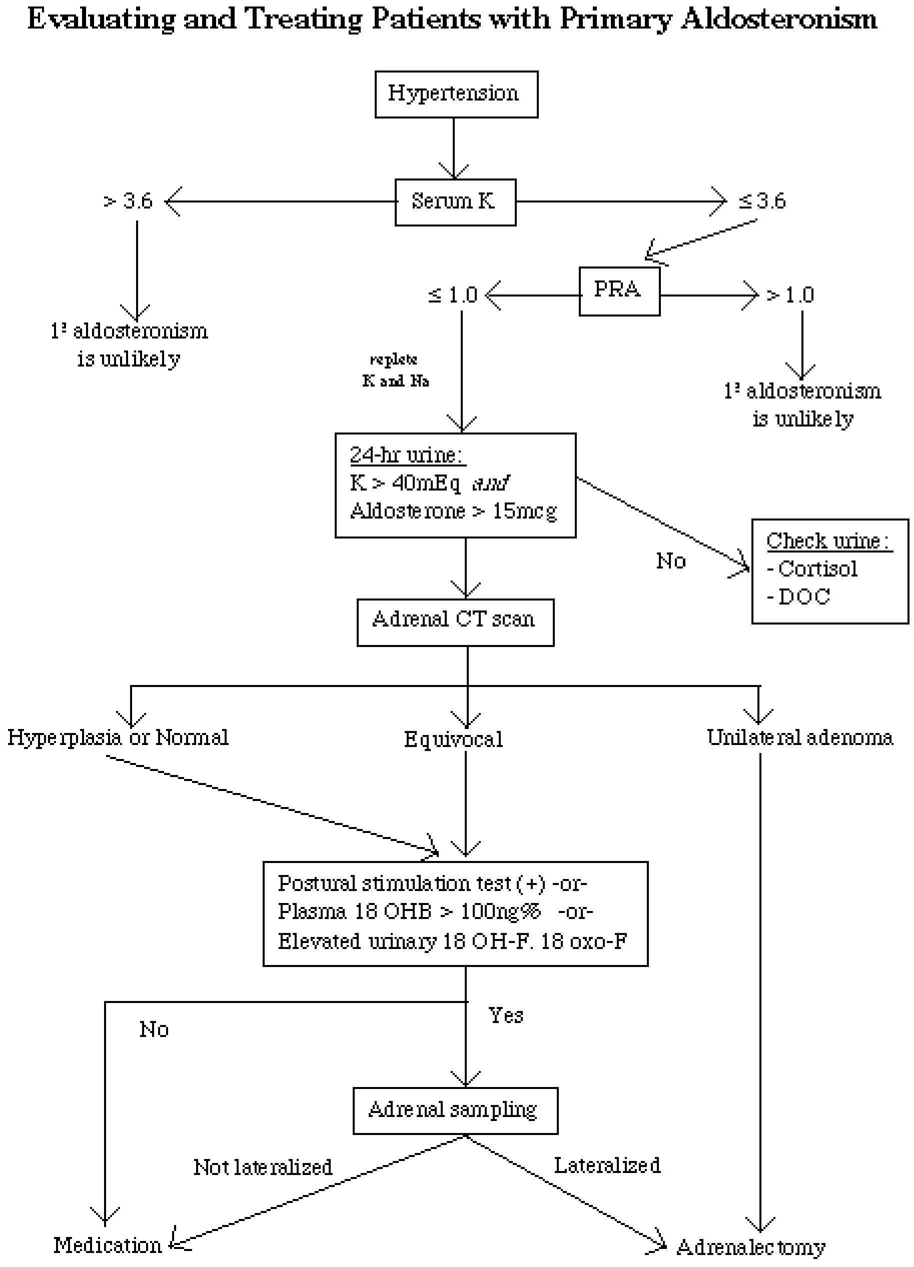

**FIGURE 5**. (From: Blumenfeld JD, Sealey JE, Schlussel Y, et al.: Dignosis and treatment of primary hyperaldosteronism. Ann Intern Med., 121:877-885, 1994.)

lateralize adrenal vein sampling for aldosterone. In this setting we have performed unilateral adrenalectomy and a significant number of those patients have favorable biochemical and blood pressure responses, although most have required the continuation of some antihypertensive medication.[26]

Finally, in the patients who have normal CT scans yet lateralize on sampling, if they show elevated 18-hydroxy products we will operate, if not we will follow those patients. The majority of patients with bilateral hyperplasia will not lateralize with adrenal vein sampling for aldosterone and those patients are treated with spironolactone at an appropriate dose to control blood pressure and often they will need other medications such as calcium channel blockers (Table 2).

### PHEOCHROMOCYTOMA

Pheochromocytoma is an uncommon entity but one which has potentially lethal sequelae for the patient if not diagnosed. Therefore, it is generally felt that all patients with sustained hypertension should have the appropriate studies performed to rule out pheochromocytoma (Fig. 6).[29]

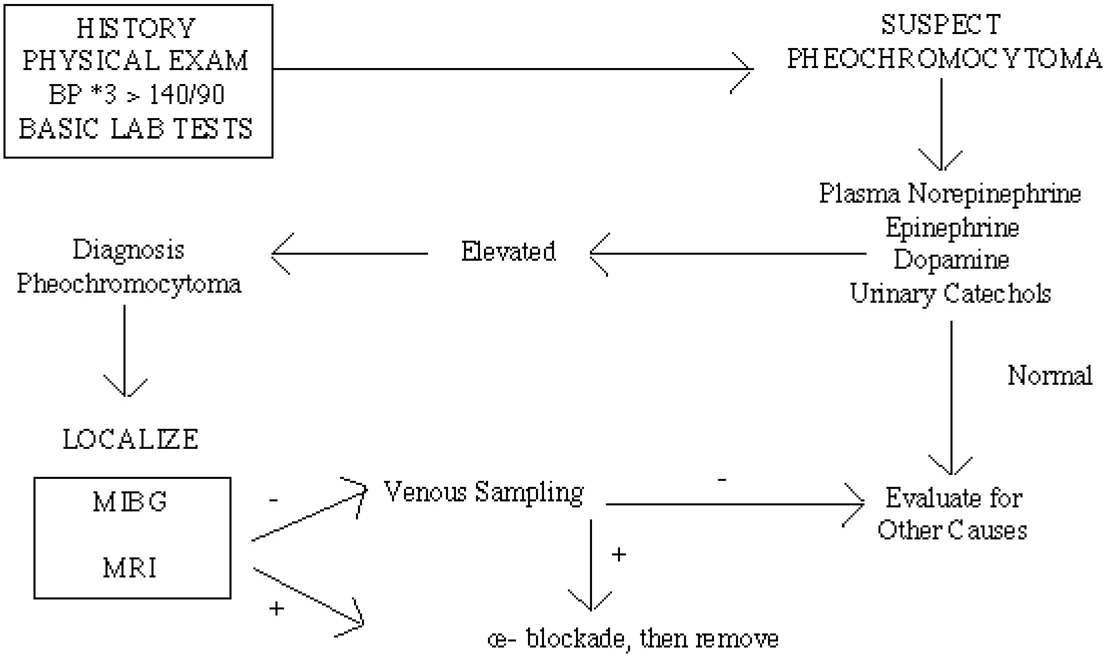

**FIGURE 6.** Evaluation for pheochromocytoma.

The clinical manifestations exhibited by patients with a pheochromocytoma are due to the physiological effects of the catecholamines, dopamine, epinephrine and norepinephrine. However other signs and symptom complexes exhibited may be extremely variable including the asymptomatic patient whereby a lesion is picked up simply on CT scan. In all reported series hypertension is by far the most common sign (Table 8). As far as the type of hypertension, the patients may either have sustained hypertension, paroxysmal or dramatic attacks of hypertension as well as sustained hypertension with superimposed paroxysms. Most series have shown this latter constellation of findings to be the most common in patients with pheochromocytoma. In addition the frequency of attacks among patients is quite variable ranging from a few times a year to multiple daily episodes. In addition, the duration may be minutes or hours and the nature of the attacks can vary dramatically. Most patients will exhibit a paroxysm or an episode once a week and most of the attacks will last less than an hour. Usually the attacks occur in the absence of recognizable stimuli but a number of factors particularly exercise, posture, trauma or a variety of other situations may precipitate an attack.

One specific entity is noteworthy and that is catecholamine induced myocardiopathy.[32,33] These patients will present with decreased cardiac function and congestive heart failure and it is mandatory that their cardiac status be stabilized with the use of appropriate alpha- and beta- adrenergic blocking agents as well as alpha-methylpara tyrosine (a tyrosine hydroxylase inhibitor) to cut down on catecholamine production before surgery is contemplated. Generally the cardiomyopathy is reversible, and the patients can be operated upon within weeks or months after the initial diagnosis and treatment is instituted.

An appreciable number of pheochromocytomas have been found in association with other disease entities and hereditary syndromes. These entities include the association of tumors of glomus jugulary region, neurofibromatosis, Sturge-Weber syndrome, the von Hippel-Landau, as well as the familial MEA (multi-endocrine adenopathy) syndromes. Pheochromocytomas occur in MEA-2, a triad including pheochromocytoma, medullary carcinoma of the thyroid and parathyroid adenomas (Sipple's syndrome). Pheochromocytomas may also be a part of MEA-3 which also includes medullary carcinoma of the thyroid, mucosal neuromas, thickened corneal nerves, ganglio-neuromatosis and frequently a marfanoid habitus. It is now believed that the relatives of patients with all of these syndromes should be evaluated for the presence of occult pheochromocytoma. In addition, there is a well known entity of familial pheochromocytoma whereby multiple members of the kindred will be found to have multiple lesions and all members of such families should be both screened and then followed for the appearance of these tumors. The mechanism of the increased incidence of pheochromocytomas in association with neuroendocrine dysplasias and medullary carcinoma of the thyroid may be explained by the APUD cell system of Pierce. The APUD cells

**TABLE 8**
**Symptoms Reported by 76 Patients (Almost All Adults) with Pheochromocytoma Associated with Paroxysmal or Persistent Hypertension**

| Symptoms | Percent paroxysmal (37 patients) | Percent persistent (39 patients) |
|---|---|---|
| Symptoms Presumably Due to Excessive Catecholamines or Hypertension | | |
| Headache (severe) | 92 | 72 |
| Excessive sweating (generalized) | 65 | 69 |
| Palpitations + tachycardia | 73 | 51 |
| Anxiety or nervousness (+ fear of impending death, panic) | 60 | 28 |
| Tremulousness | 51 | 26 |
| Pain in chest, abdomen (usually epigastric), lumbar regions, lower abdomen, or groin | 48 | 28 |
| Nausea + vomiting | 43 | 26 |
| Weakness, fatigue, prostration | 38 | 15 |
| Weight loss (severe) | 14 | 15 |
| Dyspnea | 11 | 18 |
| Warmth + heat intolerance | 13 | 15 |
| Visual disturbances | 3 | 21 |
| Dizziness or faintness | 11 | 3 |

| | | |
|---|---|---|
| Constipation | 0 | 13 |
| Paresthesia or pain in arms | 11 | 0 |
| Bradycardia (noted by patient) | 8 | 3 |
| Grand mal | 5 | 3 |
| Manifestations Due to Complications | | |
| Congestive heart failure + cardiomyopathy | | |
| Myocardial infarction | | |
| Cerebrovascular accident | | |
| Ischemic enterocolitis + megacolon | | |
| Azotemia | | |
| Dissecting aneurysm | | |
| Encephalopathy | | |
| Shock | | |
| Hemorrhagic necrosis in a pheochromocytoma | | |
| Manifestations Due to Coexisting Diseases or Syndromes | | |
| Cholelithiasis | | |
| Medullary thyroid carcinoma + effects of secretions of serotonin, calcitonin, prostaglandin, or ACTH-like substance | | |
| Hyperparathyroidism | | |
| Mucocutaneous neuromas with characteristic facies | | |
| Thickened corneal nerves (seen only with slit lamp) | | |
| Marfanoid habitus | | |
| Alimentary tract ganglioneuromatosis | | |
| Neurofibromatosis and its complications | | |
| Cushing's syndrome (rare) | | |
| Von Hippel-Lindau disease (rare) | | |
| Virilism, Addison's disease, acromegaly (extremely rare) | | |
| Symptoms Caused by Endroachment on Adjacent Structures or by Invasion and Pressure Effects of Metastases | | |

(From: Manger WM and Gifford RW Jr. Pheochromocytoma, Chapter 102, in: Hypertension Pathophysiology Diagnosis and Management, JH Laragh, BM Brenner (eds). Raven Press: New York, 1990.)

derived from the neural crest of the embryo sharing common ultrastructural and cytochemical features and elaborating amines by precursor uptake and decarboxylation.[34,35]

The laboratory diagnosis of pheochromocytoma is now extremely accurate utilizing the urinary plasma measurements of catecholamines and their byproducts. Extremely accurate assays exist for these amines.[36] At the present time it is felt that urinary catecholamines remain the measurement of choice with the measurement of total urinary catecholamines and metanephrines. Approximately 95% of patients will have elevated levels of these substances. In the patient with a severe paroxysmal hypertension who presents in the midst of hypertensive crisis, the plasma catecholamines are almost always elevated and can be utilized.

Stimulation or suppression tests are generally not utilized at the present time. The one situation where they may be useful is in the patient who appears to have essential hypertension but borderline elevated catecholamines and in this setting a clonidine suppression test may be useful. Following a single 0.3 mg oral dose of clonidine the patients with neurogenic hypertension at rest show a fall in norepinephrine whereas patients with pheochromocytomas do not.[36]

The radiographic test which is most useful in both identifying and characterizing neuroendocrine adrenal tumors, and in identifying surrounding structures is the MRI scan. We've been impressed with the multiple use of MR scans in patients with pheochromocytoma. Therefore the test is as accurate as a CT scan in identifying lesions and also has a characteristic bright lightbulb appearance on the T2 weighted study. In addition sagittal and chronal imaging can be of excellent anatomical information concerning the relationship between the tumor and the surrounding vasculature as well as draining venous channels. Therefore we feel that the MRI should be initial scanning procedure in patients with the biochemical findings of pheochromocytoma.

An alternative approach that also is useful at times, particularly for residual or multiple pheochromocytomas, is the metaiodobenzylguanidine (MIBG) scan that images medullary tissue.[37,38] Thus this test may be more sensitive than CT or MRI in picking up small extra-adrenal lesions and has its major use in patients where multiple lesions are suspected.

In summary, extremely accurate tests are available to diagnose all the major adrenal disorders.........are available if the investigator is careful and pitfalls are avoided (Table 1).

### ADRENAL SURGERY

Adrenalectomy is the treatment of choice in most patients who have undergone appropriate metabolic evaluation and have been found to have a surgical lesion. However, the surgeon must be aware that there are unique aspects to the care in these patients including specific pre-operative management as outlined in Table 2. Accordingly, patients with hyperaldosteronism who are generally healthy require spironolactone 100-400 mg/day to restore their potassium supply. Patients with Cushing's Syndrome have severe systemic effects from the hyperglucocorticoidism. They are often obese, have diabetic tendencies, are poor wound healers, easily sustain bony fractures and are susceptible to infection. Thus, they are at a high risk for complications. In selected patients with markedly elevated cortisol levels the pre-operative use of metabolic blockers such as metyrapone is required to reverse some of the clinical findings prior to adrenalectomy. Certainly glucocorticoid replacement is required throughout the surgical procedure and post-operatively until the function of the contralateral adrenal gland occurs. Finally, in patients with a pheochromocytoma, adrenergic blockade generally with dibenzylene is required and at times the blockade of catecholamine production with metyrosine is also useful as previously discussed as well as the use of other drugs (Table 9)[2]. The additional pre-operative evaluation that is mandatory in patients with pheochromocytoma is consultation with the anesthesiologist who should be well aware of the patient and can plan strategy for management.[39]

Thus the management of patients with an adrenal disorder is approached on a team basis including experienced endocrinologists, radiologists, anesthesiologists and urologists or general surgeons.

Numerous approaches can be made to the adrenal gland (Table 3). The proper approach depends on the underlying cause of adrenal pathology, the size of the adrenal, the side of the lesion, the habitus of the patient and the experience and preference of the surgeon. In addition to the surgical options a laparoscopic approach can be utilized, particularly for smaller adrenal tumors. In most cases there are a number of different option available and a careful review of all the variables is required before a choice is made. Thus each case should be considered individually although some approaches are preferable for a given disease. For example, in patients with large adrenal tumors a thoracoabdominal approach is often utilized. In contrast a posterior or modified posterior approach is preferred for small localized lesions.

**TABLE 9**
**Drug Treatment for Pheochromocytoma**

| Drug | Action | Indication | Advantage | Disadvantage |
|---|---|---|---|---|
| Phenoxybenzamine | Noncompetitive, nonselective i-blocker | All patients | Long duration Efficacious | Tachycardia, Hypotension |
| Phentolamine | Competitive i-blocker | Hypertensive crisis | Rapid onset, I.V. | Bolus can cause hypotension |
| Metoprolol, atenolol | $ß_1$-blocker | Persistent tachycardia Myocardial ischemia | $ß_1$-selective, less likely to increase BP or impair caridac function; I.V. or oral. | Hypertension crisis if incomplete i-blockade |
| Labetalol | Combined i- and ß-adrenoceptor blocker | Persistent tachycarida Myocardial ischemia | I.V. or oral. Combined blockade | Hypertensive crisis if insufficient i- blockade |
| i- methylparatyrosine | Tyrosine hydroxylase inhibitor. Reduces catecholamine biosynthesis | Cardiomyopathy, Refractory to phenoxybenzamine | Reduces catecholamine levels | Extrapyramidal side effects, crystalluria, |
| Captopril | ACE inhibitor | Heart failure, hypertension | Afterload reduction BP control | Hypotension when combined with i-blocker |
| NaCl (IV or tablets) | Volume expansion | Postural hypotension on i-blocker | Optimize volume status before surgery | Edema, volume overload |

(From: Blumenfeld JD and Vaughan Ed Jr: Hypertensive adrenal disorders, In: Therapy in Nephrology and Hypertension, Brady H and Wilcox CS (Eds.). W.B. Saunders (In Press).

Finally a patient with multiple lesions, either extra-adrenal or bilateral will be explored using a transabdominal chevron incision. Potential operative complications are shown on Table 4.

Before describing the specific techniques a number of unifying concepts warrant attention. First, adequate visualization is imperative, the adrenal glands lie high in the retroperitoneum and quite posterior. Therefore the use of a headlight by both the surgeon and first assistant is critical and hemostasis should be rigorously maintained. The operator should bring the adrenal down by initially exposing the cranial attachments and dividing the rich blood supply between either right-angled clips or utilizing a forcep cautery. Thus, it is often simplest to begin the dissection laterally, identifying the vascular supply and working around the cranial edge of the gland. The posterior surface is generally devoid of vasculature and after the gland is freed superiorly with gentle traction on the kidney, the gland can be brought inferiorly for control of the adrenal vein. The only tumor which is handled in a different fashion would be a pheochromocytoma where intent should be made to obtain control of the adrenal vein early so as to stabilize the patient from a burst of catecholamine release during manipulation. The adrenal gland is extremely friable and fractures easily which can cause troublesome bleeding. Therefore tension or traction should be maintained on the kidney or surrounding structures and not on the adrenal itself. The concept has been stated that the "patient should be dissected from the tumor", a view which is particularly true in patients for a pheochromocytomas in which the glands should not be manipulated.

### POSTERIOR APPROACH

The posterior approach can be used for either bilateral adrenal exploration or unilateral removal of small tumors. The bilateral approach is rarely utilized today because of our excellent localization techniques. It is now utilized primarily for ablative total adrenalectomy. The options for incisions are shown in the Figure; generally rib resection is preferable to gain high exposure. After standard subperiosteal rib resection, care should be taken with the diaphragmatic release, and the pleura should be avoided, and the diaphragm swept cranially.

The fibrofatty tissue within Gerota's fascia are swept away from the paraspinal musculature, exposing a subdiaphragmatic "open space" that is at the posterior apex of the resection. The liver within the periotoneum is dissected off the anterior surface of the adrenal and the cranial blood supply is divided. Medially on the right, the inferior vena cava (IVC) is visualized. The short, high adrenal vein entering the cava in a dorsolateral position is identified and can be clipped or ligated. The adrenal can then be drawn caudally by traction on the kidney. The adrenal arteries will issue from under from behind the inferior vena cava and these must be carefully clipped or otherwise troublesome bleeding can occur.

Finally the adrenal is removed from the superior aspect of the kidney and care must be taken to avoid apical branches of the renal artery. On the left, the approach is similar with division of the splenorenal ligament giving initial lateral exposure.

The posterior approach can be modified for a transthoracic adrenal exposure to the diaphragm;[40] however, this more extensive approach is rarely necessary for small adrenal tumors.

### MODIFIED POSTERIOR APPROACH

Although the posterior approach has the advantage of rapid adrenal exposure and low morbidity, there are definite disadvantages. This approach may impair respiration, the abdominal contents are compressed posteriorly, and the visual field is limited. In addition, if bleeding occurs and it is difficult to extend the incision to gain a better exposure. Therefore, we have developed a modified posterior approach for right adrenalectomy utilizing the Gil-Vernet position.[41]

The approach is based on the anatomical relationship with the right adrenal which lies deeply posterior and high in the retroperitoneum behind the liver (Fig. 7). In addition, the short, stubby right adrenal vein enters the IVC posteriorly at the apex of the adrenal. Hence, we utilize an approach that is posterior, but the patient is in a modified position, similar to that used for a Gil-Vernet dorsal lumbotomy incision (Fig. 8).[42] The patient is first placed in a formal lateral flank position and then allowed to fall forward into the modified posterior position. Subsequently the eleventh or twelfth rib is resected with care to avoid the pleura. The diaphragm then is dissected off the underlying peritoneum and liver in order to gain mobility. Similarly, the inferior surface of the peritoneum, closely associated with the liver, is sharply dissected from Gerota's fascia, which is gently retracted inferiorly. It is of note that the adrenal gland is not identified during the early portion of the dissection and because of the modified posterior approach, the surgeon can become disoriented if not thoroughly familiar with anatomical relationships.

The adrenal will become visible in the depth of the incision as the final hepatic attachments are divided. The lateral, empty space can be found exposing the posterior abdominal musculature and often the inferior vena cava. Multiple small arterial course behind the inferior vena cava, emerge over the paraspinal muscles and these are clipped and divided.

At this point the adrenal can usually be moved posteriorly against the paraspinal muscles exposing the anterior surface of the inferior vena cava below the adrenal gland.

The major advantage of this approach is that the adrenal vein is easily identified because it emerges from the segment of the IVC exposed and courses up to the adrenal, which now rises toward the surgeon. In other flank or anterior positions the adrenal vein resides in its posterior relationship, requiring caval rotation and the chance of adrenal vein avulsion. After adrenal vein exposure, it is doubly tied and divided or clipped with right-angled clips and divided.

The remaining removal of the adrenal is as was previously described for the posterior approach.

On the left side we do not use this modified approach and use a standard flank approach with a fairly small incision.

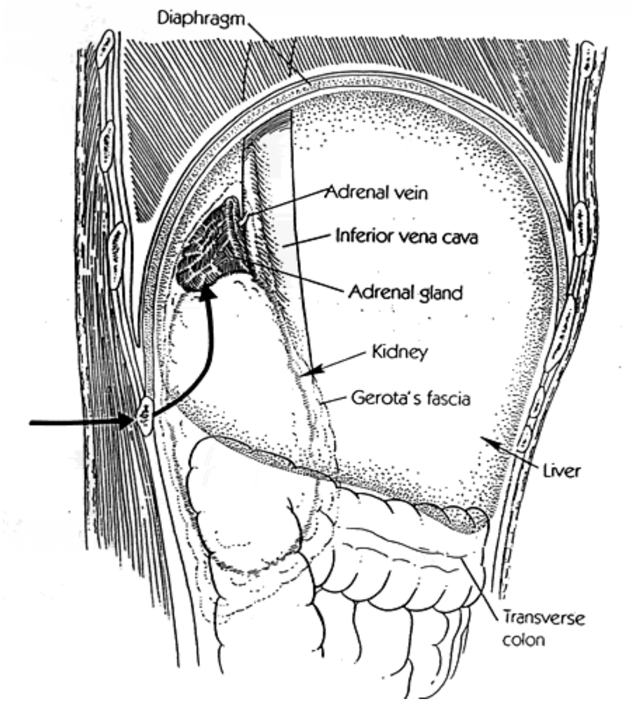

**FIGURE 7**. Anatomical relationship of right adrenal and liver.

We have used the modified posterior approach for all patients with right adrenal aldosterone-secreting tumors and for other patients with benign adenomas of less than 6 cm. We do not recommend the approach for patients with large lesions or malignant adrenal neoplasms. The approach has been used for patients with relatively small pheochromocytomas.

### FLANK APPROACH

The standard extrapleural, extraperitoneal eleventh rib resection is excellent for either left or right adrenalectomy. After completion of the incision, the lumbocostal arch is utilized as a landmark showing the point of attachment of the posterior diaphragm to the posterior abdominal musculature. Gerota's fascia, containing the adrenal and kidney, can be swept medially and inferiorly, giving exposure to the splenorenal ligament on the left, which should be divided to avoid splenic injury (Fig. 8). Working anteriorly on the left, the spleen and pancreas within the peritoneum can be lifted cranially, exposing the anterior surface of the adrenal gland.

On the right side, similar maneuver is used to lift the liver within the peritoneum off the anterior surface of the adrenal. Quite often the adrenal gland cannot be identified precisely until these maneuvers are performed. One should not attempt to dissect into the body of the adrenal or to dissect the inferior surface of the adrenal off the kidney. The kidney is useful for retraction. The dissection should continue from lateral to medial along the posterior abdominal and diaphragmatic musculature, with precise ligation or clipping of the small but multiple adrenal arteries. While the operator clips these arteries with one hand, the opposite hand is employed to retract both adrenal and kidney inferiorly (Fig. 9). With release of the superior vasculature, the adrenal becomes easily visualized. On the left medially, the phrenic branch of the venous drainage must be carefully clipped or ligated (Fig. 2). This vessel is not noted in most atlases but can cause troublesome bleeding if divided. The medial dissection along the crus of the diaphragm and aorta will lead to the renal vein; finally, the adrenal vein is controlled, doubly tied, and divided. The adrenal is then removed from the kidney with care to avoid the apical branches of the renal artery.

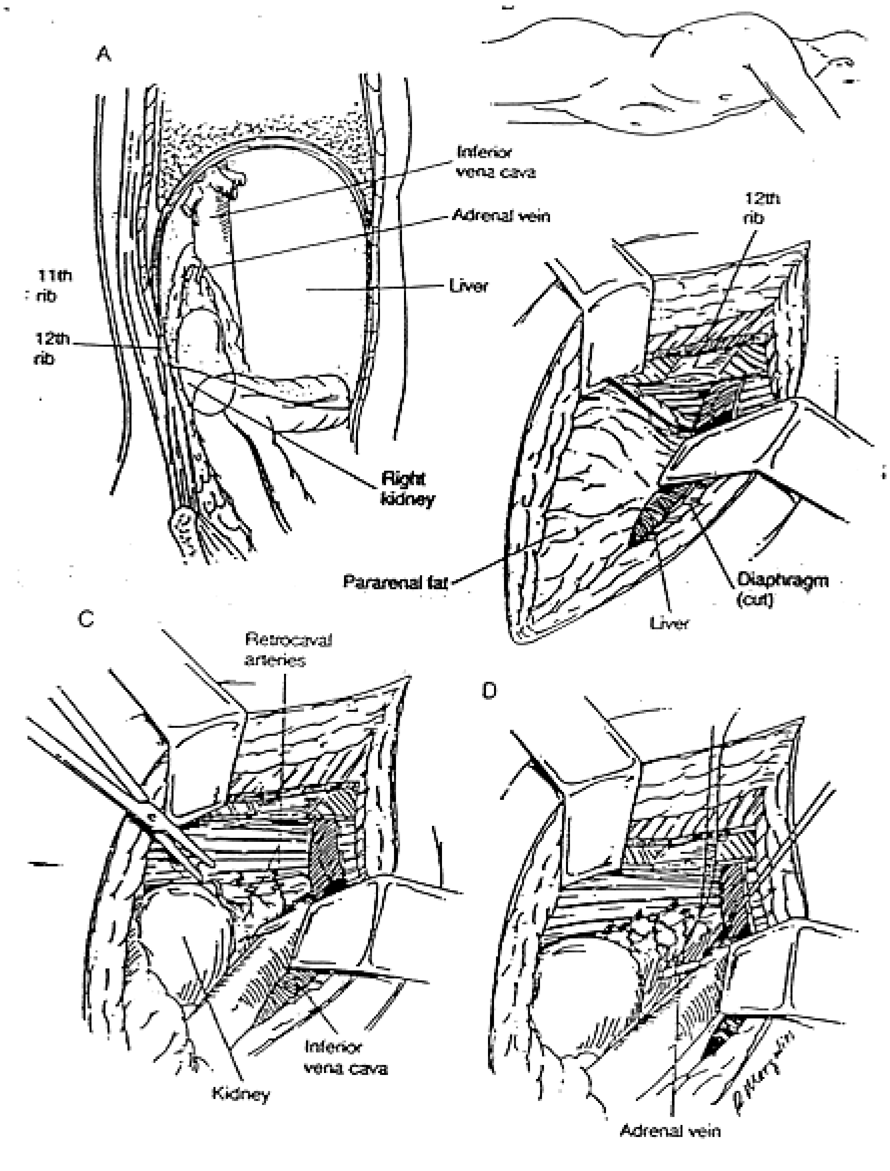

**FIGURE 8**. Exposure of the right adrenal vein from the modified posterior approach. If the vein is injured an Alis clamp can be placed at its origin and the cava oversewn.

On the right side, the dissection is similar. However, after release of the adrenal from the superior vasculature, it is helpful to expose the IVC and divide the medial arterial supply. This maneuver allows mobilization of the cava for better exposure of the high posterior adrenal vein, which is doubly tied or clipped and divided. Patients with large adrenal carcinomas may require en bloc resection of the adrenal and kidney following the principles of radical nephrectomy (Fig. 10).

A major deviation from this technique is used for the patient with pheochromocytoma, in whom the initial dissection should be aimed toward early control and division of the main adrenal vein on either side. Obviously, in this setting, the anesthesiologist should be notified when the adrenal vein is divided because a marked drop in blood pressure often occurs, even when the patient is adequately hydrated.

After removal of the adrenal, inspection should be made for any bleeding and for pleural tears of the diaphragm. The kidney should also be inspected. The incision is closed without drains with interrupted 0 polydioxanone sutures.

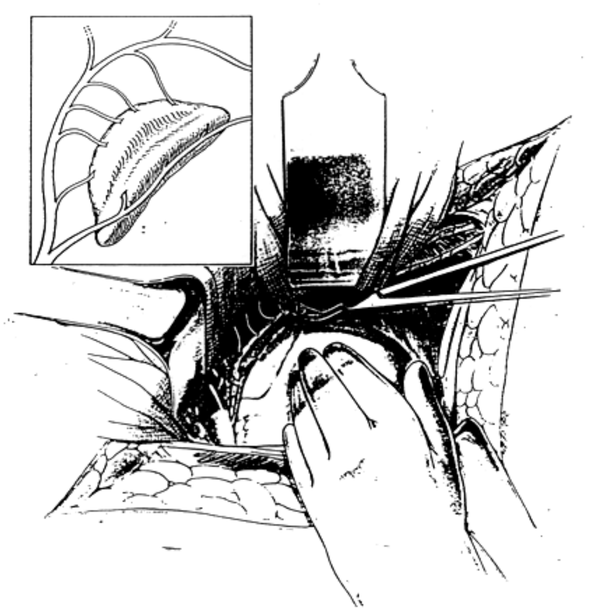

**FIGURE 9**. Cephalad exposure of the left adrenal with careful division of arterial supply.

### THORACOABDOMINAL APPROACH

The thoracoabdominal ninth or tenth rib approach is utilized for large adenomas; for some large adrenal carcinomas; and for well-localized pheochromocytomas. The incision and exposure is standard, with a radial incision through the diaphragm and a generous intraperitoneal extension. The techniques described for adrenalectomy with the eleventh rib approach are used.

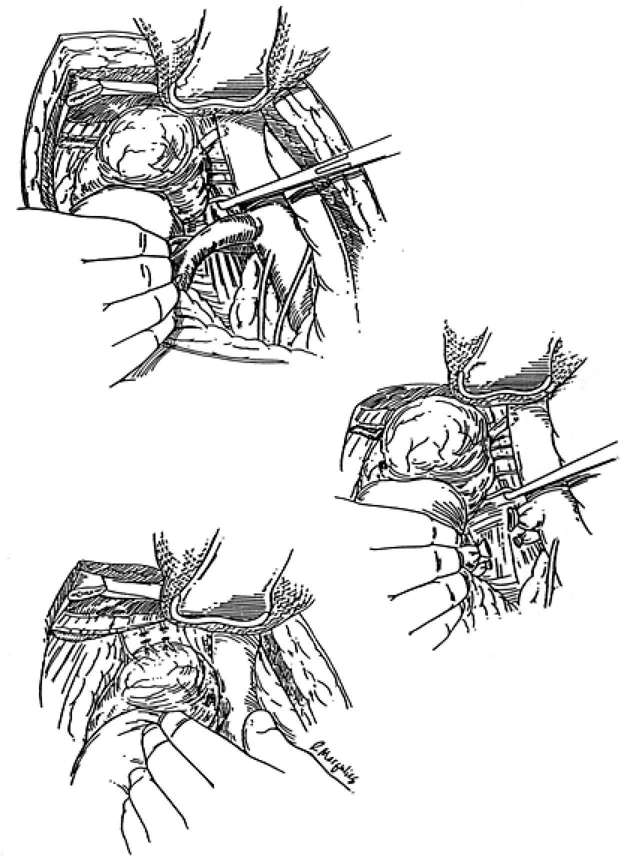

**FIGURE 10.** Removal of large right adrenal tumor and kidney.

### TRANSABDOMINAL APPROACH

The transabdominal approach is commonly selected for patients with pheochromocytomas, for children, and for some patients with adrenal carcinomas. The concept is to have the ability for complete abdominal exploration to identify either multiple pheochromocytomas or adrenal metastases.

I use the transverse or chevron incision, which I believe gives better exposure of both adrenal glands than a midline incision. The rectus muscles and lateral abdominal muscles are divided, exposing the peritoneum. Upon entering the peritoneal cavity, the surgeon should gently palpate the para-aortic areas and the adrenal areas. Close attention is given to blood pressure changes in an attempt to identify any unsuspected lesions if the patient has a pheochromocytoma. This maneuver is less important today because of the excellent localization techniques previously discussed. In fact, with precise pre-operative localization of the offending tumor, the chevron incision does not need to be completely symmetrical and may be limited on the contralateral side.

If the patient has a lesion on the right adrenal, the hepatic flexure of the colon is reflected inferiorly. The incision is made in the posterior peritoneum lateral to the kidney and carried superiorly, allowing the liver to be reflected cranially (Fig. 11). Incision in the peritoneum is carried downward, exposing the anterior surface of the to the entrance of the right renal vein. Once the cava is cleared, one or two accessory hepatic veins are often encountered, which should be secured. These veins are easily avulsed from the cava and may cause troublesome bleeding. Ligation of these veins gives 1 to 2 cm of additional caval exposure, which is often useful during the exposure of the short posterior right adrenal vein. Small accessory adrenal veins may also be encountered. The cava is then rolled medially, exposing the adrenal vein, which should be doubly tied or clipped and divided.

After control of the adrenal vein, it is simplest to proceed with the superior dissection, lifting the liver off the adrenal and securing the multiple small adrenal arteries arising from the inferior phrenic artery, which is rarely seen. The adrenal can be drawn inferiorly with retraction on the kidney, and the adrenal arteries traversing to the adrenal from under the cava can be secured with right angled clips. The final step is removing the adrenal from the kidney.

The left adrenal vein is not as difficult to approach because it lies lower, partially anterior to the upper pole of the kidney, and the adrenal vein empties into the left renal vein. Accordingly, on the left side, the colon is reflected medially, exposing the anterior surface of Gerota's capsule; the initial dissection should involve identification of the renal vein. In essence, the dissection is the same as for a radical nephrectomy for renal carcinoma. Once the renal vein is exposed, the adrenal vein is identified, doubly ligated, and divided. After this maneuver the pancreas and splenic vasculature are lifted off the anterior surface of the adrenal gland. Because of additional drainage from the adrenal into the phrenic system, I generally continue the medial dissection early to control the phrenic vein. I then work cephalad and lateral to release the splenorenal ligament and the superior attachments of the adrenal. The remainder of the dissection is carried out as previously described.

After removal of the tumor, regardless of size, careful inspection is made to ensure hemostasis and the absence of injury to adjacent organs. Careful abdominal exploration is carried out, after which the wound is closed with the suture material of choice. No drains are used.

Patients with multiple endocrine adenopathy or family histories of pheochromocytoma, as well as pediatric patients, should be considered at high risk for multiple lesions. Pre-operative evaluation should identify these lesions, but, regardless, a careful abdominal exploration should be carried out.

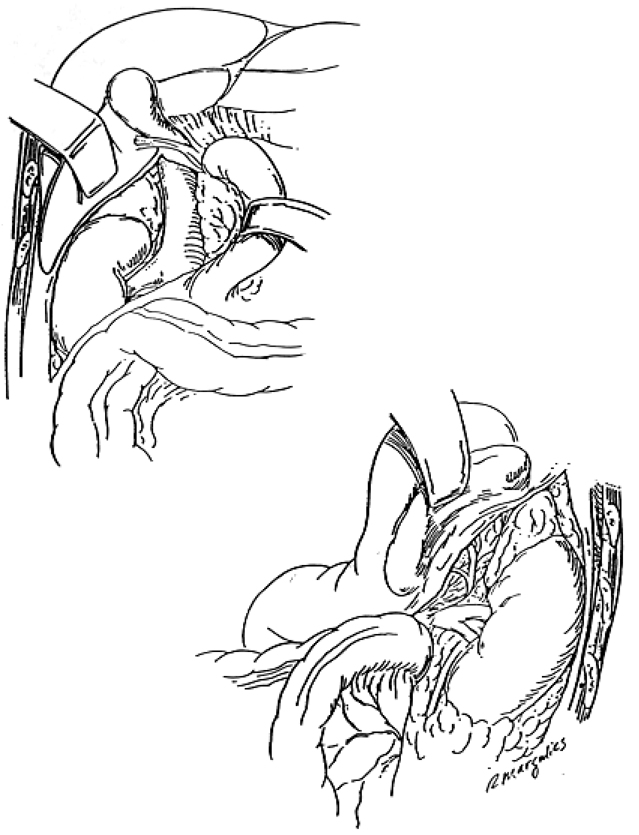

**FIGURE 11.** Relationship of the adrenal to intra-abdominal organisms. Care should be taken to avoid injury to the spleen, splenic vein and pancreas on the left and the liver and duodenum on the right.

In patients with suspected malignant pheochromocytomas, en bloc dissections may be necessary to obtain adequate margins, a concept that also applies in patients with adrenal carcinomas. Evaluation with MRI to obtain transverse, coronal, and sagittal images is extremely useful to define clearly the adrenal relationships to the IVC and renal vessels as well as to localize the adrenal vein.

In patients with pheochromocytomas, post-operative management includes maintenance of arterial and venous lines in an intensive care setting until they are stable. Often, 24 to 48 hours is required for the full effect of phenoxybenzamine, the alpha-blocking agent commonly given, to wear off and for normal alpha-receptor activity to be restored (Table 5).

### PARTIAL ADRENALECTOMY

The standard treatment for patients with the adrenal lesions described has been total adrenalectomy. However, there recently has been reported an excellent paper showing the utility of partial adrenalectomy in patients with primary hyperaldosteronism.[43] I have not used partial adrenalectomy in a patient for normal contralateral adrenal, but certainly have used the technique in patients with bilateral disease. Thus, in one patient with a pheochromocytoma on one side and a non-functioning adenoma on the other, the adenoma was simply enucleated from the adrenal. In a second patient with bilateral pheochromocytomas, the larger lesion was totally excised and partial adrenalectomy was utilized to remove the contralateral tumor. Care has to be made to obtain thorough hemostasis when performing a partial adrenalectomy because of the vascular nature of the adrenal.

### LAPAROSCOPIC ADRENALECTOMY

The techniques of laparoscopic surgery are discussed elsewhere in this text. There is an expanding literature which is now available, describing both the surgical technique and the results of laparoscopic adrenalectomy.[43-45] It is abundantly clear that the technique is feasible, is safe and is well tolerated by the patient. One of the major limitations of the widespread usefulness of the procedure is the fact that a coordinated and well experienced laparoscopic team need to be available to perform the procedure. In addition, even with an experienced team, the time required to perform a laparoscopic unilateral adrenalectomy far exceeds the time required for an open procedure and as of yet, there is little cost advantage to performing the procedure other than the fact that the patient can return to work at an earlier time. The specific technical points for the procedure are described elsewhere and are not incorporated into this discussion.

### SUMMARY

We are fortunate that our ability to diagnose the specific adrenal entities which mandate a surgical approach is extremely accurate. The combination of analytical methodology to measure the appropriate adrenocortical and medullary hormonal production and the radiologic techniques for localization are superb. The management of these adrenal disorders with precise surgical precision following localization is highly successful, resulting in a reversal of both metabolic abnormalities and the hypertension which often accompanies these diseases. Indeed this is a true success story with the evolution of these different techniques over the past 50 years.

### REFERENCES

1. Vaughan ED Jr: Adrenal tumors, Chapter In: Urologic Oncology, JP Richie, JE Oesterling (Eds.). W.B. Saunders Co.,

1998 (In press).
2. Blumenfeld JD and Vaughan ED Jr: Hypertensive adrenal disorders, In: Therapy in Nephrology and Hypertension, Brady H and Wilcox CS (Eds.). W. B. Saunders (In press).
3. Vaughan ED Jr: Complications of adrenal surgery, In: Complications of Urologic Surgery, 3rd Edition. SS Taneja, RM Ehrlich and RB Smith (Eds.). W.B. Saunders Co. (In press).
4. Vaughan ED Jr and Blumenfeld JD: The Adrenals, Chapter 96 In: Campbell's Urology, 7th Edition. PC Walsh, AB Retik, TA Stamey, and ED Vaughan Jr (Eds.). W.B. Saunders Co.:Philadelphia, pp. 2915, 1998.
5. Lee MJ, Mayo-Smith WW, Hahn PF, Goldberg MA, Boland GW, Saini S, and Papanicolaou N: State-of-the-art MR imaging of the adrenal gland. RadioGraphics, 14:1015-1029, 1994.
6. Vaughan ED Jr: Adrenal surgery, Chapter 24, In: Textbook of Operative Urology. FF Marshall (Ed.). W.B. Saunders Co.: Philadelphia, 1996.
7. Orth DN: Cushing's Syndrome. N Eng J Med., 32:791, 1995.
8. Kazam E, Engel IA, Zirinsky K, Auh JH, Rubenstein WA, Reckler JA and Markisz JA: Sectional imaging of the adrenal glands, computed tomography and ultrasound, In: Adrenal Disorders, ED Vaughan Jr, and RM Carey (Eds.). Thieme Medical Publishers Inc: New York, 1989.
9. Belldegrun A, and deKernion JB: What to do about the incidentally found adrenal mass. World J Urol., 7:117-120, 1989.
10. Hedican SP, and Marshall FF: Cardiac bypass and postoperative mitotane in the treatment of adrenocortical carcinoma with caval extension. Proceedings of the American Urological Association, Abstract #751, Vol. 155, pg. 498A, 1996.
11. Nakajo M, Nakabeppu Y, Yonekura R, Iwashita S, Goto T: The role of adrenocortical scintigraphy in the evaluation of unilateral incidentally discovered adrenal and juxtaadrenal masses. Annls of Nucl Med., 7(3):157-166, 1993.
12. Ross NS, and Aron DC: Hormonal evaluation of the patient with an incidentally discovered adrenal mass. N Engl J Med., 323:1401, 1990.
13. Cerfolio RJ, Vaughan ED Jr, Brennan TG, Hiruela ER: Accuracy of computed tomography in predicting adrenal tumor size. Surg Gyn & Obstet, 176:307, 1993.
14. Mayo-Smith WW, Lee MJ, McNicholas MM, Hahn PF, Boland GW and Saini S: Characterization of adrenal masses (<5cm) by use of chemical shift MR imaging: Observer performance vs. Quantitative measures. AJR, 165:91-95, 1995.
15. Lubat E, and Weinreb JC: Magnetic resonance imaging of the kidneys and adrenals. Top Magn Reson Imag., 2:17-36, 1990.
16. Tikkakoski T, Taavitsainen M, Paivansalo M, Lahde S, Apaja-Sarkkinen M: Accuracy of adrenal biopsy guided by ultrasound and CT. Acta Radiologica, 32:371-374, 1991.
17. Luton J-P, Cerdas S, Billaud L, Thomas G, Guilhaume B, Bertagna X, Laudat M-H, Louvel A, Chapuis Y, Blondeau P, Bonnin A, and BricaireH: Clinical features of adrenocortical carcinoma, prognostic factors, and the effect of mitotane therapy. N Engl J Med., 322:1195, 1990.
18. Imperato-McGinley J, Young IS, Huang T, Dreyfus JC III, Reckler JM and Peterson RE: Testosterone secreting adrenal cortical adenomas. Int J Gynaecol Obstet., 19:421, 1981.
19. Gabrilove JL, Sharma DC, Waitz HH, and Dorfman R: Feminizing adrenal cortical tumors in the male: A review of 52 cases including a case report. Medicine, 44:37, 1965.
20. Richie JP and Gittes RF: Carcinoma of the adrenal cortex. Cancer, 45:1957, 1980.
21. Pommier RF and Brennan MF: An eleven-year experience with adrenocortical carcinoma. Surgery, 112:963-971, 1992.
22. Haak HR, van Seters AP, Moolenaar AJ, Fleuren GJ: Expression of p-glycoprotein in relation to clinical manifestation, treatment and prognosis of adrenocortical cancer. Eur J Cancer, 29A:1036-1038, 1993.
23. Bergenstal DM, Hurtz R, Lipsett MB and Moy RH: Chemotherapy of adrenal cortical cancer with o,p DDD. Ann Intern Med., 53:672, 1960.
24. Wooten MD, and King DK: Adrenal cortical carcinoma. Epidemiology and treatment with mitotane and a review of the literature. Cancer, 72:3145-3155, 1993.
25. Conn JW: Primary hyperaldosteronism. A new clinical syndrome. J Lab Lin Med., 45:3, 1955.
26. Blumenfeld JD, Schlussel Y, Sealey JE, et al.: Diagnosis and treatment of primary hyperaldosteronism. Ann Int Med., 121:877-885, 1994.
27. Laragh JH, Angers M, Kelly WG, et al.: The effect of epinephrine, norepinehphrine, angiotensin II, and others on the secretory rate of aldosterone in man. JAMA, 174:234, 1960.
28. Laragh JH, and Sealey JE: The renin-angiotensin-aldosterone system and the renal regulation of sodium, potassium, and blood pressure homeostasis. In: Handbook of Physiology, EE Windhager (Ed.). Oxford University Press: New York, 1992.
29. Vaughan ED Jr and Sosa RE: Renovascular hypertension, Chapter 11 In: Campbell's Urology, 7th Edition. PC Walsh, AB Retik, TA Stamey, and ED Vaughan Jr (Eds.). W.B. Saunders Co.:Philadelphia, pp. 423-459, 1998.
30. Mann SJ and Atlas SA: Hypertensive emergencies, Chapter 181 In: Hypertension, Pathophysiology, Diagnosis and Management, JH Laragh, BM Brenner (Eds.). Raven Press: New York, 1995.
31. Manger WM, and Gifford RW Jr: Pheochromocytoma: A clinical review, Chapter 132, In: Hypertension, Pathophysiology, Diagnosis and Management, JH Laragh, BM Brenner (Eds.). Raven Press: New York, 1995.

32. Imperato-McGinley J, Gautier T, Ehlers K, Zullo MA, Goldstein DS and Vaughan ED Jr: Reversibility of catecholamine-induced dilated cardiomyopathy in a child with a pheochromocytoma. N Engl J Med., 316:793-797, 1987.
33. Vaughan ED Jr.: Catecholamine cardiomyopathy. In: Clinical and Experimental Pheochromocytoma, Second Edition. WM Manger, RW Gifford Jr. Blackwell Science Publications: Cambridge. Chap. 8, pp. 421-422, 1996.
34. Bolande RP: The neurocrestopathias: A unifying concept of disease arising in neurocrest maldevelopment. Hum Pathol., 5:409, 1974.
35. Pearse AG, and Polak JM: Cytochemical evidence for the neural crest origin of mammalian ultimobranchial C cells. Histochemie, 27:96, 1971.
36. Bravo EL: Pheochromocytoma: New concepts and future trends. Kid Intl., 40:544-556, 1991.
37. Shapiro B, Copp JE, Sisson JC, Ayer TL, Wallis J, and Beierwaltes WH: Iodine-131 metaiodobenzylguanidine for the locating of suspected pheochromocytoma: Experience in 400 cases. J Nucl Med., 26:576, 1985.
38. Campeau RJ, Garcia OM, Correa OA, Rege AB: Pheochromocytoma: Diagnosis by scintigraphy using iodine-131 metaiodobenzylguanidine. Southern Med J., 84:1221-1230, 1991.
39. Malhotra V (Editor): Anesthesia for Renal and Genitourinary Surgery, McGraw Hill: New York, 1995.
40. Novick AC, Straffon RA, and Kaylor W: Posterior transthoracic approach for adrenal surgery. J Urol., 141:254, 1989.
41. Vaughan ED Jr and Phillips H: Modified posterior approach for right adrenalectomy. Surg Gynecol Obstet., 165:453-455, 1987.
42. Gil-Vernet J: New surgical concepts in removing renal calculi. Urol Int., 20:255-262, 1965.
43. Nakada T, Kubota Y, Sasagawa I, Yagisawa T, Watanabe M, Ishigooka M: Therapeutic outome of primary aldosteronism: Adrenalectomy versus enucleation of aldosterone-producing adenoma. J Urol., 153:1775-1780, 1995.
44. Suzuki K, Kageyama S, Ueda D, Ushiyama T, Kawabe K, Tajima A, and Aso Y: Laparoscopic adrenalectomy: Clinical experience with 12 cases. J Urol., 150:1099-1102, 1993.
45. Naito S, Uozumi J, Shimura H, Ichimiya H, Tanaka M, and Kumazawa J: Laparoscopic adrenalectomy: Review of 14 cases and comparison with open adrenalectomy. J of Endourol., 9:491-495, 1995.
46. MacGillivray DC, Shichman SJ, Ferrer FA, Malchoff CD: A comparison of open versus laparoscopic adrenalectomy. Surgical Endoscopy, 10:987, 1996.

**This article should be referenced as follows:**

Vaughan, E.D., Jr. and Colt, J.J. (2004) Surgery of the adrenals. *TheScientificWorldJOURNAL* **4 (S1)**, 400–426.

**Handling Editor:**

Anthony Atala, Principle Editor for *Urology* — a domain of *TheScientificWorldJOURNAL*.
